# Insulin signaling pathways in lepidopteran ecdysone secretion

**DOI:** 10.3389/fphys.2014.00019

**Published:** 2014-02-05

**Authors:** Wendy A. Smith, Anthony Lamattina, McKensie Collins

**Affiliations:** Department of Biology, Northeastern UniversityBoston, MA, USA

**Keywords:** insulin, ecdysteroids, *manduca sexta*, insects, prothoracic gland, prothoracicotropic hormone

## Abstract

Molting and metamorphosis are stimulated by the secretion of ecdysteroid hormones from the prothoracic glands. Insulin-like hormones have been found to enhance prothoracic gland activity, providing a mechanism to link molting to nutritional state. In silk moths (*Bombyx mori*), the prothoracic glands are directly stimulated by insulin and the insulin-like hormone bombyxin. Further, in *Bombyx*, the neuropeptide prothoracicotropic hormone (PTTH) appears to act at least in part through the insulin-signaling pathway. In the prothoracic glands of *Manduca sexta*, while insulin stimulates the phosphorylation of the insulin receptor and Akt, neither insulin nor bombyxin II stimulate ecdysone secretion. Involvement of the insulin-signaling pathway in *Manduca* prothoracic glands was explored using two inhibitors of phosphatidylinositol-3-kinase (PI3K), LY294002 and wortmannin. PI3K inhibitors block the phosphorylation of Akt and 4EBP but have no effect on ecdysone secretion, or on the phosphorylation of the MAPkinase, ERK. Inhibitors that block phosphorylation of ERK, including the MEK inhibitor U0126, and high doses of the RSK inhibitor SL0101, effectively inhibit ecdysone secretion. The results highlight differences between the two lepidopteran insects most commonly used to directly study ecdysteroid secretion. In *Bombyx*, the PTTH and insulin-signaling pathways intersect; both insulin and PTTH enhance the phosphorylation of Akt and stimulate ecdysteroid secretion, and inhibition of PI3K reduces ecdysteroid secretion. By contrast, in *Manduca*, the action of PTTH is distinct from insulin. The results highlight species differences in the roles of translational regulators such as 4EBP, and members of the MAPkinase pathway such as ERK and RSK, in the regulation of insect ecdysone secretion, and in the impact of nutritionally-sensitive hormones such as insulin in the control of ecdysone secretion and molting.

## Introduction

Secretion of the steroid hormone, ecdysone, from the insect prothoracic glands triggers molting and metamorphosis. Insect insulin-like hormones have been implicated in the regulation of prothoracic gland activity, linking nutritional state and ecdysis. As in vertebrates, insect insulin-like hormones activate a tyrosine-kinase-linked receptor, resulting in the activation of phosphatidylinositol kinase (PI3K), protein kinase B/Akt (Akt), and target-of-rapamycin (TOR). TOR enhances protein synthesis through the phosphorylation and inactivation of an inhibitory binding protein for initiation factor 4E (4EBP), and the phosphorylation and activation of ribosomal S6 kinase. In *Drosophila*, genetic manipulation of the insulin-signaling pathway strongly implicates insulin-like hormones in prothoracic gland activity. In *Drosophila*, overexpression of PI3K increases gland size and transcription of ecdysone target genes, as well as the steroidogenic genes *phantom* and *dib* (Caldwell et al., 2005; Colombani et al., 2005; Mirth et al., 2005). Overexpression of PTEN, a lipid phosphatase that counteracts the effects of PI3K, or expression of dominant negative PI3K have the converse effect. Treatments that specifically increase glandular size through insulin signaling result in premature metamorphosis and small adults, while those that reduce glandular size lead to extended feeding and an increase adult body size, suggesting that the prothoracic glands, through response to insulin, serve as size-sensors in developing insects (Caldwell et al., 2005; Colombani et al., 2005; Mirth et al., 2005).

Insulin-like hormones have been found to increase ecdysone secretion by isolated prothoracic glands from the silk moth *Bombyx mori* (Kiriishi et al., 1992; Gu et al., 2009), and the bug, *Rhodnius prolixus* (Vafopoulou and Steel, 1997). However, ecdysone secretion by the prothoracic glands in *Manduca* is not stimulated by insulin (Walsh and Smith, 2011). Nonetheless, *Manduca* prothoracic glands appear to be insulin-responsive: insulin stimulates autophosphorylation of the insulin receptor (IR) and the phosphorylation of Akt, and prothoracic glands from nutritionally deprived *Manduca* show changes in the insulin-signaling pathway including upregulation of the IR (Walsh and Smith, 2011). Further, feeding of the TOR inhibitor rapamycin leads to smaller prothoracic glands, mimicking nutritional deprivation and delaying molting (Kemirembe et al., 2012).

In the present study, we further pursued the signaling pathways stimulated by insulin in *Manduca* to identify possible differences in the effects of insulin-like hormones on ecdysone secretion, focusing on probable sites of cross-talk between insulin-like hormones and PTTH. Like insulin, PTTH stimulates a tyrosine-kinase linked receptor. The PTTH receptor is known as Torso, first characterized in *Drosophila* embryos (Casanova and Struhl, 1989; Li, 2005; Rewitz et al., 2009). Unlike insulin, PTTH increases intracellular levels of cyclic AMP, through a PTTH-stimulated increase in intracellular calcium and the activation of a calcium-sensitive adenylyl cyclase (Smith et al., 1984, 1985; Meller et al., 1988; Dedos et al., 2005, 2007; Fellner et al., 2005). Increased intracellular calcium also results in activation of mitogen-activated protein kinases (MAPkinases) including MEK and ERK (Rybczynski and Gilbert, 2003). Inhibition of MEK effectively blocks PTTH-stimulated ecdysone secretion in *Bombyx* and *Manduca* (Rybczynski and Gilbert, 2003; Gu et al., 2010). In *Drosophila*, inactivation of the MAPkinase signaling pathway inhibits prothoracic gland activity and the action of Torso (Caldwell et al., 2005; Rewitz et al., 2009).

Protein synthesis is a key feature of prothoracic gland activation by PTTH. PTTH enhances translation, and inhibitors of translation block PTTH-stimulated ecdysone secretion (Keightley et al., 1990; Gilbert et al., 1997). In keeping with an effect of PTTH on translation, the phosphorylation of a 34 kDa protein characterized as ribosomal protein S6 is stimulated by PTTH, calcium, or cAMP analogs (Smith et al., 1986; Song and Gilbert, 1994, 1995). Similarly, in vertebrates, protein synthesis is required for ecdysone secretion (Keightley et al., 1990; Stocco and Clark, 1996). In vertebrates, a critical translation-dependent event is the synthesis of a short-lived protein known as steroidogenic acute regulatory protein (StAR). StAR activates cholesterol transfer from the outer to the inner mitochondrial membrane, enhancing steroid hormone synthesis (Stocco and Clark, 1996). Proteins with START1 domains, homologous to vertebrate StAR, have been found in insects, though their roles in ecdysone secretion are not clear (Roth et al., 2004; Sakudoh et al., 2005). It is likely that cholesterol mobilization of some type also underlies insect steroid hormone secretion (Lafont et al., 2011).

Studies by Gu et al. have characterized cross talk between the PTTH and insulin pathways in *Bombyx mori*. In this insect, PTTH stimulates the phosphorylation of Akt, suggestive of the activation of PI3K by PTTH (Gu et al., 2011b, 2012). Insulin, after 8 h exposure, also stimulates ecdysone secretion, particularly after the 4th day of the last larval instar. Inhibition of PI3K with wortmannin or LY294002 reduces basal and insulin- or PTTH-stimulated secretion. Inhibition of ecdysone secretion is associated with reduction in the phosphorylation of 4EBP, suggesting reduced protein synthesis. Further, the inhibition of ecdysone secretion by PI3K inhibitors occurs without a reduction in the phosphorylation of ERK, indicating that PTTH in *Bombyx* calls into play two required signaling pathways (Gu et al., 2011a).

The present study was undertaken to delineate points of intersection in *Manduca* prothoracic glands between PTTH and insulin. The results highlight a critical role for MAPkinases in Manduca in ecdysone secretion by a pathway distinctly independent from that stimulated by insulin.

## Materials and methods

### Animals

M. *sexta* eggs were obtained from Carolina Biological Supply (Burlington, NC) or from adults raised from this stock. Larvae were reared on an artificial diet (Bell, 1976) at 25°C under a photoperiod of 16 h-light/8 h-dark. Feeding fifth instar larvae were used in experiments (4–7 g, days 2–3).

### Reagents and hormones

Grace's insect culture medium was obtained from Invitrogen. LY294002 and wortmannin, obtained from BioMol, U0126 from Calbiochem, and SL0101 from Toronto Research Chemicals, were prepared as stock solutions in DMSO and diluted in Grace's for use in experiments. Phosphopeptide and secondary antibodies were obtained from Cell Signaling Technology (phosphoAkt *Drosophila* Ser505; phosphoIGF-1receptor Tyr1135/1136; phosphoRSK; phospho4EBP; HRP-labeled anti-rabbit or anti-mouse secondary antibody), or Santa Cruz Biotechnology (phosphoERK). The location of these proteins in the insulin- and PTTH-signaling pathways is diagrammed in Figure 9.

Recombinant PTTH and synthetic bombyxin II were generous gifts of Drs. Hiroshi Kataoka and Dr. Shinji Nagata (University of Tokyo). Human recombinant insulin solution was used (Sigma, 10 mg/ml), diluted into Grace's medium. *Manduca* brain extracts were prepared in our lab, using frozen day 0 pupal brains. Because we were using a crude extract, brains were initially homogenized in acetone to remove compounds that might interfere with the radioimmunoassay. This is the same first step used in preparing *Bombyx* brains for subsequent purification of bombyxin (Nagasawa et al., 1984). The homogenate was briefly centrifuged at 1000 × g and the pellet was then subjected to extraction by homogenizing in cold 2% NaCl, cooling on ice, centrifuging for 10 min at 10,000 × g, again, similar to the initial preparation of *Bombyx* bombyxins. The pellet was extracted one additional time in 2% NaCl, and the combined supernatants stored at −20°C. Insulin-like hormones can multimerize (see for example, Pandyarajan and Weiss, 2012), so to retain as broad a spectrum of insulin-like proteins as possible, we chose not to subject the homogenate to further filtration, as would be done to isolate PTTH (Walsh and Smith, 2011). Instead centrifugation was used to clarify the extract, as was done for preparations of bombyxin-containing brain extracts by Nijhout et al. (2007).

### Prothoracic gland incubations and ecdysone radioimmunoassays

Larval prothoracic glands were dissected into lepidopteran saline and maintained in Grace's medium for periods of less than 1 h prior to experimentation. Individual glands were pre-incubated in standing droplets of culture medium, with or without hormones or inhibitors, for indicated periods of time. Experiments were terminated by placing glands directly into 2X SDS-sample buffer for Western blotting, described below. Medium was removed and stored at −20°C for ecdysone RIAs, conducted as previously described (Warren et al., 1984). The ecdysone antibody was produced in rabbits against an ecdysone-22-succinyl thyroglobulin synthesized by Dr. D.H.S. Horn (C.S.I.R.O., Canberra, Australia).

### Western blots

Larval prothoracic glands were incubated in test or control solutions for the designated incubation periods, placed into SDS-sample buffer, and boiled for 3–5 min. Details of Western blot procedures are the same as those described previously (Smith et al., 2003). Samples were run on 8 or 13.5% SDS-PAGE gels to separate proteins. The samples were then transferred from gels to nitrocellulose membranes at 4°C for 75–90 min. The membranes were incubated with primary antibody overnight, then rinsed and treated with appropriate secondary antibody for 75 min, rinsed again, and bands visualized with Western blotting luminol reagents (Pierce ECL Western blotting substrate). The blots were exposed on chemiluminescence film (Marsh BioProducts Blue Film, carried by AbGene) and developed (Kodak GBX fixer and developer).

### Statistics

Statistical analysis was performed using a One-Way ANOVA with a Tukey-Kramer *post-hoc* test to compare specific differences. For the insulin and bombyxin stimulation experiments, a paired *t*-test was used. In all cases, *p* < 0.05 was used to determine significance.

## Results

**Figure 1:**
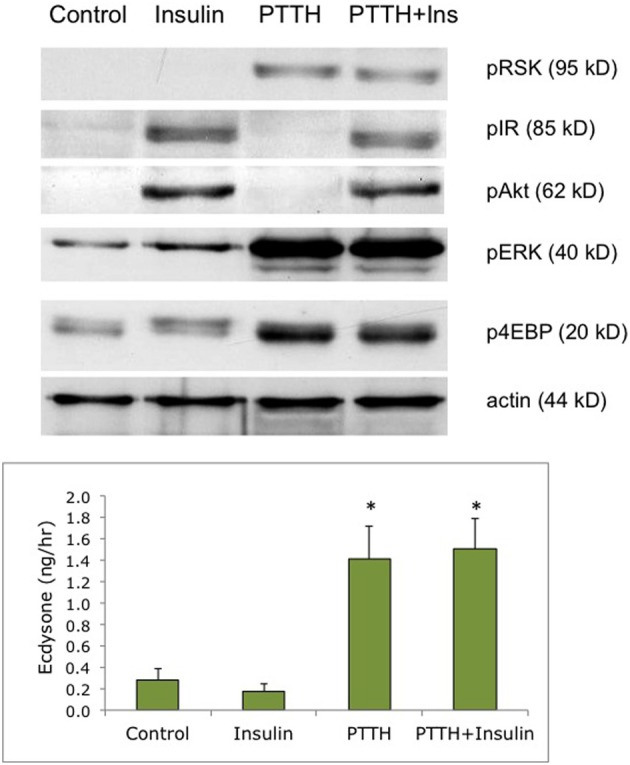
**Effects of PTTH and insulin on signaling phosphoproteins**. Prothoracic glands from day 2 fifth instar larvae were incubated for 20 min medium with or without insulin (6 uM), followed by 80 min in one of the following treatments: no hormone (Control), insulin (6 uM), PTTH (7 nM), or insulin + PTTH. (TOP) Western blots of cellular lysates, probed for phosphoproteins; (BOTTOM) Ecdysone secretion determined by radioimmunoassay of culture medium at the end of the 80 incubation period. Asterisks denote statistically significant ecdysone secretion relative to no-hormone controls (*n* = 5 glands per group). Insulin did not enhance secretion, alone or in conjunction with PTTH (ANOVA, Tukey–Kramer *post-hoc* test, significance *p* > 0.05).

**Figure 2:**
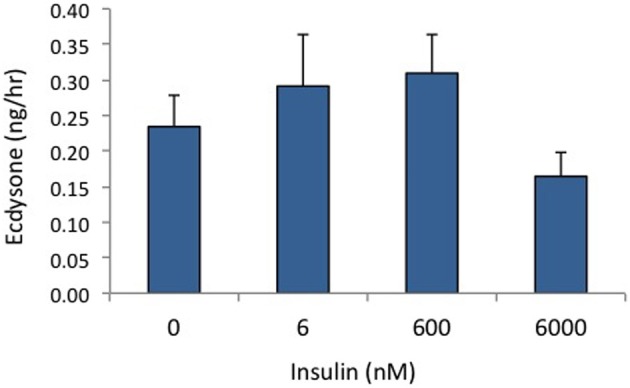
**Effects of insulin on ecdysone secretion**. Prothoracic glands from day 2 fifth instar larvae were incubated for 80 min in designated concentrations of insulin, and culture medium was assayed for ecdysone (*n* = 9–14 glands per group). None of the insulin-treated groups differed from controls (ANOVA, Tukey–Kramer *post-hoc* test, *p* > 0.05).

We have shown in previous experiments that *Bombyx* bombyxin II and *Manduca* brain extract stimulate the phosphorylation of signaling proteins in the insulin pathway in developing wing discs, including the phosphorylation of IR and Akt (Nijhout et al., 2007; Walsh and Smith, 2011). In the present study, we compared phosphoproteins stimulated by insulin with those stimulated by recombinant PTTH in the prothoracic glands. We used as our experimental tissue prothoracic glands removed from day 2–3 of the fifth instar, when the glands are sensitive to PTTH but not yet secreting high levels of steroid. As shown in Figure 1, insulin and PTTH elicit distinctly different patterns of phosphoprotein activation. PTTH enhances the phosphorylation of RSK, ERK, and 4EBP. By contrast, insulin enhances the phosphorylation of the IR and Akt. Ecdysteroid assays indicate that insulin does not stimulate ecdysteroid secretion on its own, at doses ranging from 6 nM to 6 uM (Figure 2), nor does it augment the effects of PTTH (Figure 1). Previous experiments had shown that insulin does not stimulate glands removed from day 5 (wandering) larvae (Walsh and Smith, 2011).

**Figure 3:**
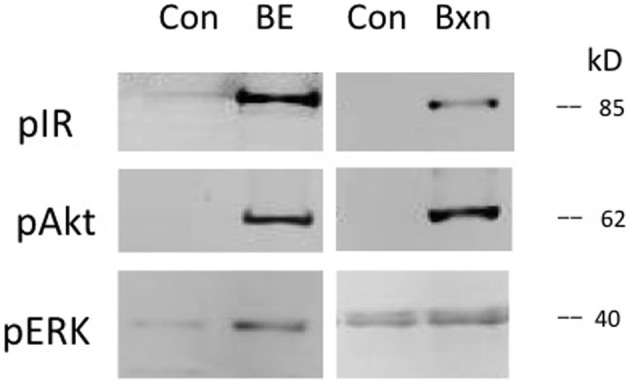
**Effects of *Manduca* brain extract and *Bombyx* bombyxin II on signaling phosphoproteins**. Prothoracic glands from day 2 fifth instar larvae were incubated for 30 min in Grace's medium (Control), medium containing crude *Manduca* brain extract (BE, 0.5 brain equivalents/30 ul) with both PTTH and insulin-like activities, or medium containing synthetic *Bombyx* bombyxin II (Bxn, 160 nM). At the end of the incubation period cellular lysates were probed for phosphoproteins via Western blots.

**Figure 4:**
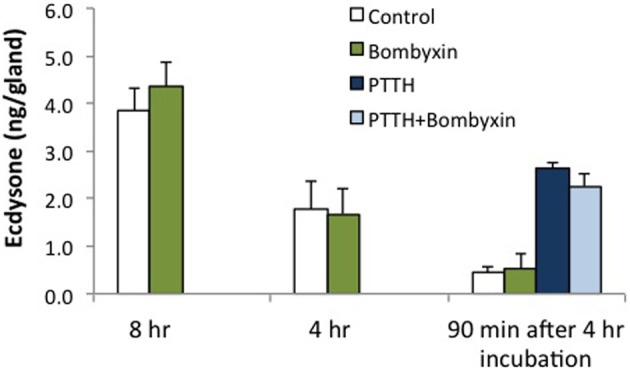
**Effects of *Bombyx* bombyxin II on ecdysone secretion**. Prothoracic glands from day 3 fifth instar larvae were incubated for 4 or 8 h in medium containing no hormone (Control) or *Bombyx* bombyxin II (160 nM) (*n* = 6 and 11 glands per group, respectively). In separate experiments, glands were incubated for 4 h in no hormone or 160 nM bombyxin, and then challenged for 90 min in medium with no hormone, bombyxin, PTTH (7 nM) or PTTH + bombyxin (*n* = 16 glands per group). Culture medium was assayed for ecdysone by radioimmunoassay. None of the bombyxin-treated groups differed from their respective controls (ANOVA, Tukey–Kramer *post-hoc* test, *p* > 0.05).

**Figure 5:**
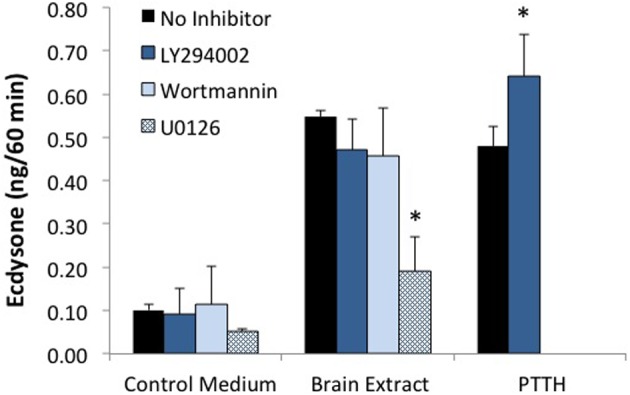
**Effects of PI3K inhibitors (LY294002 and wortmannin), and MEK inhibitor (U0126) on ecdysone secretion**. Prothoracic glands from day 3 fifth instar larvae were incubated for 20 min in medium with or without the designated inhibitor, followed by 60 min in one of the following treatments: No Inhibitor (Grace's medium); LY294002 (60 uM); Wortmannin (10 uM); U0126 (10 uM); Brain Extract (0.5 brain equivalents/30 ul); Brain Extract + LY294002; Brain Extract + Wortmannin; Brain Extract + U0126; PTTH (7 nM); or PTTH + LY294002. At the end of the 60 min incubation period, culture medium was assayed for ecdysone by radioimmunoassay. Ecdysone secretion by groups treated with U0126 was significantly lower than by groups in the respective no-inhibitor controls (asterisks) (ANOVA, Tukey–Kramer *post-hoc* test, significance *p* < 0.05). Sample sizes were as follows (glands per group): Control = 51; BE = 38; LY294002 = 36; BE + LY294002 = 27; U0126 = 4; BE + U0126 = 5; Wortmannin = 10; BE + Wortmannin = 12; PTTH = 19; PTTH + LY = 19.

Prothoracic glands were also tested with *Bombyx* bombyxin II (generously provided by Drs. Hiroshi Kataoka and Shinji Nagata). Bombyxin II is 41% identical to *Manduca* bombyxin (GenBank AAY84557.1, UniProt Q4JJX8_MANSE). Purified or recombinant forms of *Manduca* bombyxin are not currently available. Over 30 bombyxin genes have been cloned in *Bombyx* (Mizoguchi and Okamoto, 2013), hence it is likely that *Manduca* also possesses more than one type of bombyxin. We tested a crude *Manduca* brain extract, casting a wide net for *Manduca* endogenous insulin-like hormones. As shown in Figure 3, bombyxin II and *Manduca* brain extract, like insulin, stimulate phosphorylation of the IR and Akt. For this reason, we believe that the brain extract contains insulin-like activity. Because brain extract also contains PTTH, ERK is phosphorylated in response to brain extract (Figure 3), and brain extract stimulates ecdysone secretion (Figure 5). Bombyxin does not stimulate ERK phosphorylation (Figure 3), nor does it stimulate ecdysone secretion, even following prolonged exposure (Figure 4). Further, we do not observe that bombyxin increases responsiveness to PTTH (Figure 4).

**Figure 6:**
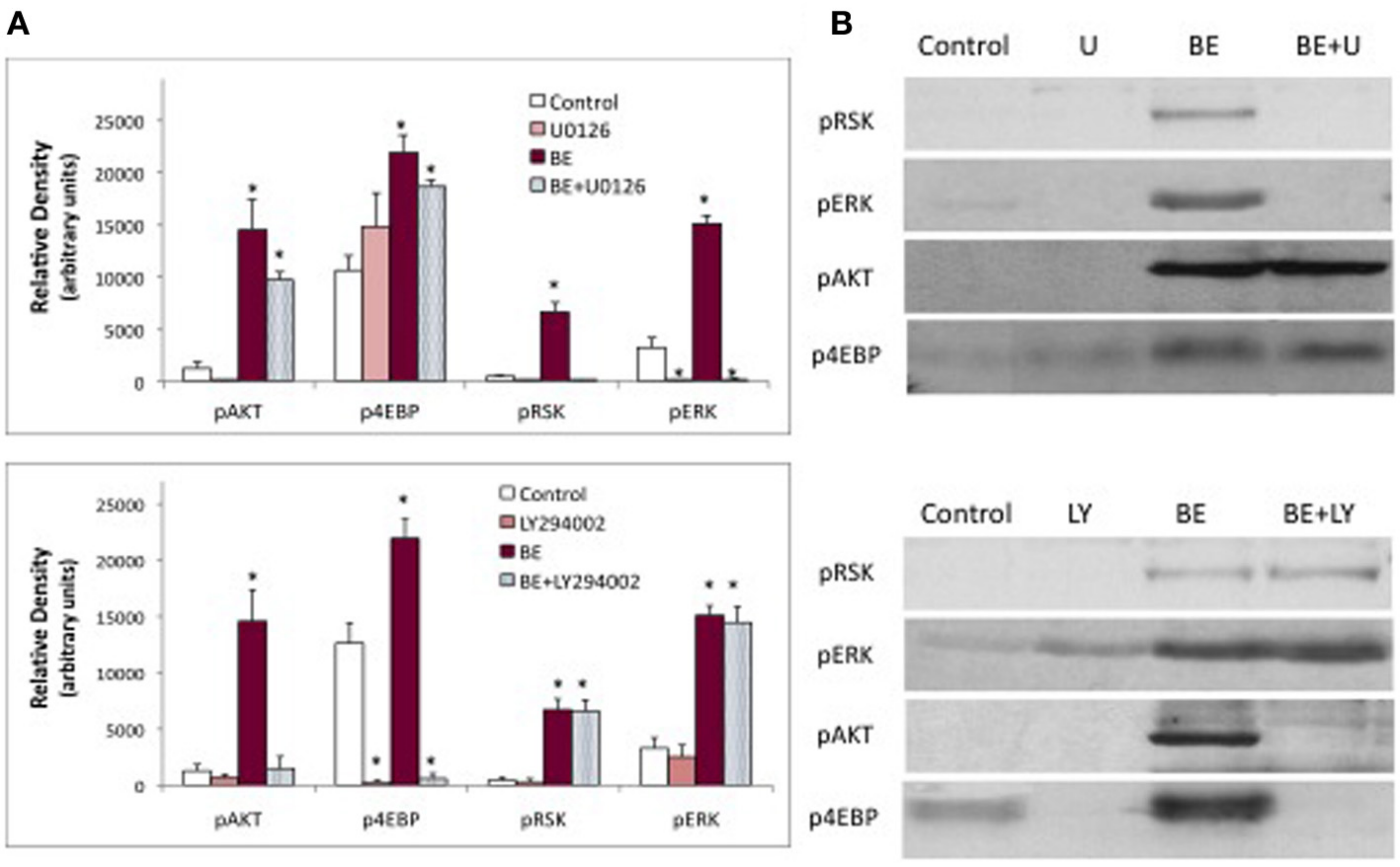
**Effects of PI3K inhibitor (LY294002) and MEK inhibitor (U0126) on protein phosphorylation**. Western blots were prepared from prothoracic glands described in Figure 5, and probed for phosphoproteins. Treatments were as follows: no hormone (Control), 10 uM U0126 (U), 60 uM LY294002 (LY), brain extract (BE, 0.5 brain equivalents/30 ul), brain extract + U0126 (BE + U) or brain extract + LY294002 (BE + LY). **(A)** Results of a typical experiment. **(B)** Quantification of Western blots. Asterisks indicate groups that were statistically different than Controls (ANOVA, Tukey–Kramer *post-hoc* test, *p* < 0.05). *n* = 4–16 samples per group.

A critical step in the action of insulin in both insects and vertebrates is the activation of PI3K. This kinase is inhibited by LY294002 or wortmannin. As seen in Figure 5, PTTH- and brain-extract-stimulated ecdysteroid secretion are unaffected by PI3K inhibitors. The efficacy of the PI3kinase inhibitors was confirmed by the absence of Akt phosphorylation (Figure 6). Hence, while brain extract does contain insulin-like ligands (evidenced by phosphorylation of the IR), if any of these ligands stimulate ecdysone secretion, neither they, nor PTTH, do so through PI3K. By contrast, the MEK (MAP kinase kinase) inhibitor U0126 inhibits basal and hormone-stimulated ecdysteroid secretion.

The effects of inhibitors on protein phosphorylation were examined in brain extract-treated samples. Because the extract contains both PTTH and insulin-like hormones, multiple phosphoproteins could be visualized including pRSK, pERK, and p4EBP (stimulated by PTTH in the extract) and pAkt (stimulated by bombyxin in the extract) (Figure 6). The MEK inhibitor U0126 blocks the phosphorylation of ERK and RSK, has no significant effect on Akt, and on its own slightly stimulates the phosphorylation of 4EBP. By contrast, the PI3K inhibitor LY294002 has no effect on the phosphorylation of ERK or RSK, but completely blocks the phosphorylation of Akt. LY294002 also blocks basal and hormone-stimulated phosphorylation of 4EBP.

**Figure 7:**
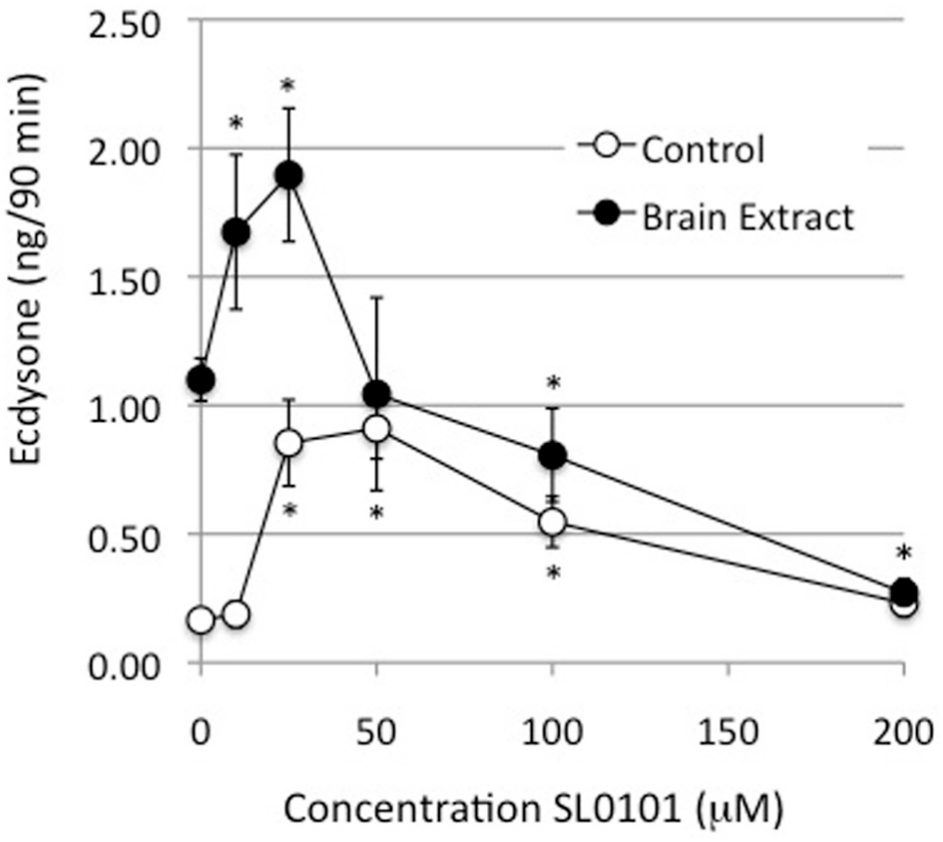
**Effects of RSK inhibitor (SL0101) on ecdysone secretion**. Prothoracic glands from day 3 fifth instar larvae were incubated for 20 min in medium with or without the designated doses of SL0101, followed by 90 min in designated doses of SL0101 with or without brain extract (0.5 Brain equivalents/30 μl). At the end of the incubation period culture medium was assayed for ecdysone by radioimmunoassay. Ecdysone secretion by groups treated with 10, 25, or 50 μM SL0101 was statistically higher than groups incubated in the absence of inhibitor. Ecdysone secretion by groups treated with brain extract, and 100 or 200 uM SL0101, was significantly lower than groups treated with brain extract in the absence of inhibitor. Asterisks = significant differences from groups incubated in the absence of inhibitor (ANOVA, Tukey-Kramer *post-hoc* test, *p* < 0.05). *n* = 8–21 glands per SL0101-treated group; Controls = 53 glands per group; BE = 53 glands per group.

**Figure 8:**
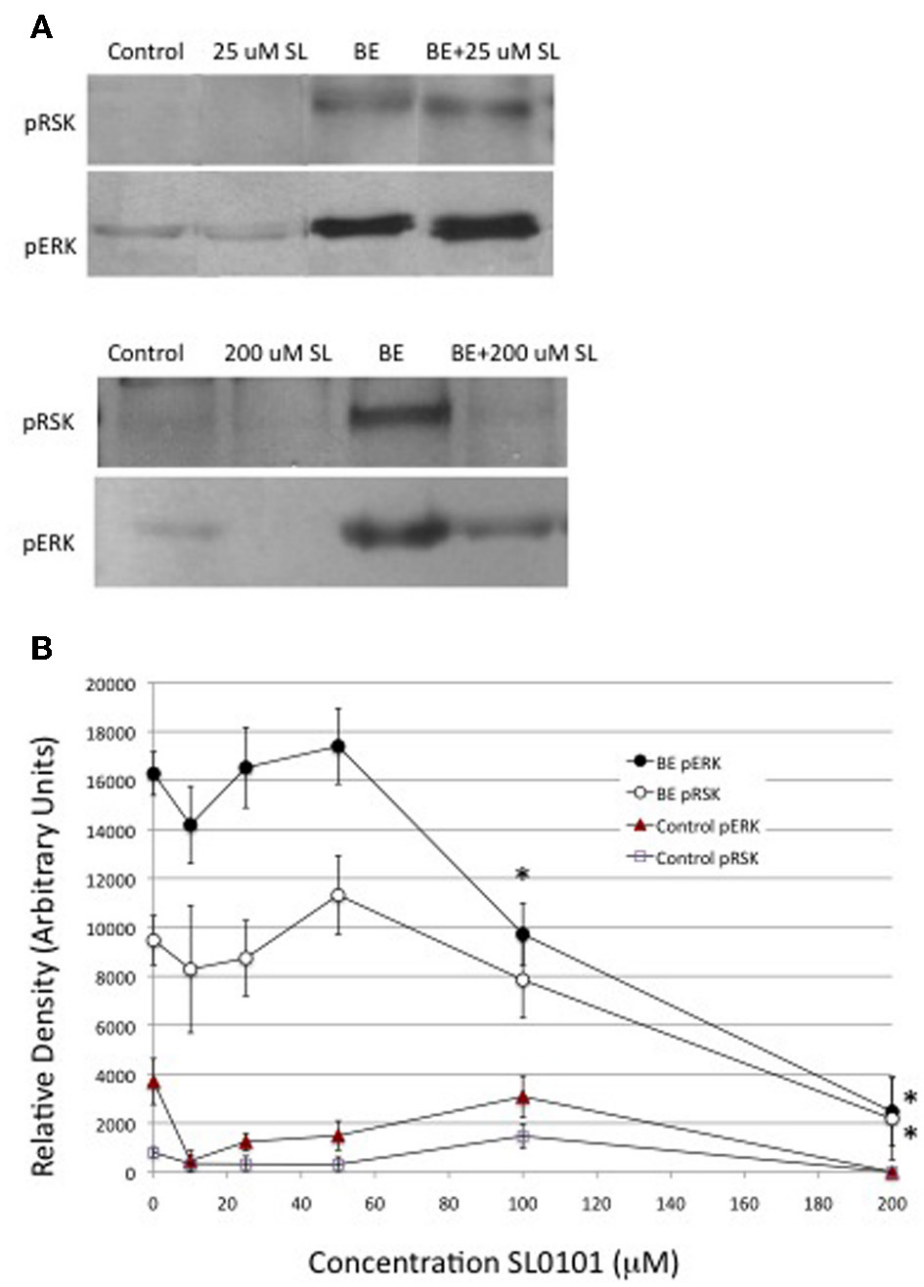
**Effects of RSK inhibitor (SL0101) on protein phosphorylation**. Western blots were prepared from prothoracic glands described in Figure 7. Cellular lysates were probed for phosphoproteins via Western blots. **(A)** Results of a typical experiment. **(B)** Quantification of Western blots. Asterisks indicate groups that were statistically greater than their respective no-hormone controls (ANOVA, Tukey–Kramer *post-hoc* test). Sample sizes were as follows (replicates per group): Control = 28; BE = 28; 10 uM SL = 2; 25 uM SL = 7; 50 uM SL = 3; 100 uM SL = 15; 200 uM SL = 2; BE + 10 uM SL = 2; BE + 25 uM SL = 10; BE + 50 uM SL = 6; BE + 100 uM SL = 13; BE +200 uM SL = 6.

The inhibition of ecdysone secretion by a MEK inhibitor, but not by a PI3K inhibitor, indicates that in *Manduca*, the MAPkinase pathway is a more critical mediator of hormone-stimulated ecdysone secretion than PI3K. An important downstream enzyme in the MAPkinase pathway is RSK (Romeo et al., 2012), hence a RSK inhibitor, SL0101, was tested for its effects on ecdysteroid secretion. SL0101 targets the kinase domain of RSK (Smith et al., 2005). The inhibitor would be expected to inhibit phosphorylation events downstream of RSK itself, which, unfortunately, we did not have a means of visualizing. We found that 10, 25, and 50 uM doses of SL0101 actually increased basal ecdysone secretion, and a 25 uM dose significantly enhanced hormone-stimulated secretion (Figure 7). The reason for a stimulatory effect of RSK inhibition is not clear. By contrast, 100 and 200 uM doses of the RSK inhibitor significantly inhibited ecdysone secretion (Figure 7). However, as shown in Figure 8, 100 and 200 uM SL0101 also significantly reduced the phosphorylation of ERK, and 200 uM SL0101 reduced the phosphorylation of RSK, suggesting that the inhibitor at high doses may be acting non-specifically as a MEK inhibitor.

## Discussion

The present study confirms a lack of direct stimulation of insulin-like hormones on *Manduca* ecdysone secretion, as seen in an earlier study (Walsh and Smith, 2011). Our results do not rule out a role for insulin in regulating *Manduca* prothoracic gland function. We have previously shown that glands from nutritionally starved larvae exhibit increased transcription of IR and cellular content of 4EBP, in keeping with a resulting enhanced sensitivity to insulin. Indeed, injection of insulin reduces total 4EBP levels in the prothoracic glands (Walsh and Smith, 2011). However, insulin injection does not increase ecdysone secretion (Walsh and Smith, 2011), and neither insulin nor bombyxin augment prothoracic gland sensitivity to PTTH. It is possible, however, that a bombyxin isoform not tested in the present study has a stimulatory effect or potentiating effect on *Manduca* prothoracic gland secretory activity. Such a factor would need to activate the prothoracic glands in some manner beyond the IR and Akt, which do not alone appear to stimulate ecdysone secretion by *Manduca* prothoracic glands.

**Figure 9:**
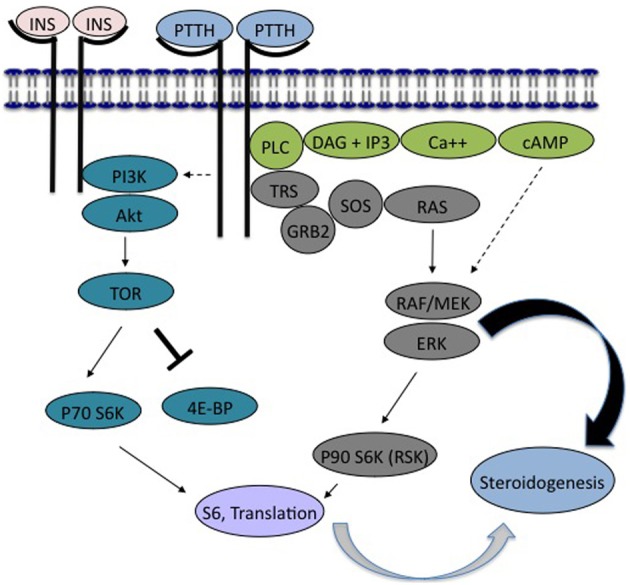
**PTTH and insulin signaling pathways in lepidopteran ecdysone secretion**. PTTH: In both *Bombyx* and *Manduca*, PTTH has been shown to stimulate tyrosine kinase activity. We suggest that this leads to the phosphorylation of a postulated receptor substrate (modeled as Torso Receptor Substrate, TRS), capable of interacting with PLC (phospholipase C). PTTH is known to increase intracellular calcium, activate calcium-sensitive adenylyl cyclase (not shown), and to elevate cyclic AMP. TRS is postulated to recruit the adapter protein GRB2 and SOS, enhancing the activity of RAS and RAF, leading to known activation of MEK, ERK, and RSK. Cyclic AMP is also suggested to act via an effect on the MAPkinase pathway (dotted line). Insulin/Bombyxin: In both Bombyx and Manduca, insulin or bombyxin activate PI3K and Akt, which is presumed to increase the activity of TOR (target of rapamycin) through proteins not shown. TOR enhances translation by suppressing the translation inhibitor 4E-binding protein (4EBP), and activating p70 S6 kinase, which targets ribosomal protein S6. The primary difference seen between the prothoracic glands of Manduca and Bombyx is the ability of both insulin and PTTH to activate the insulin signaling pathway in Bombyx (PI3K, Akt, TOR, 4EBP) and by this pathway to stimulate ecdysone secretion (Gray curved arrow). By contrast, in Manduca, although PTTH stimulates the phosphorylation of 4EBP suggesting activation of PI3K (dotted line), the insulin signaling pathway is neither sufficient, nor required, for ecdysone synthesis. Instead, secretion is mediated by PTTH-stimulated activation of MAPkinases (Black curved arrow).

In *Bombyx*, insulin does directly stimulate ecdysone secretion, as do high doses of bombyxin (Kiriishi et al., 1992; Gu et al., 2009). Further, unlike *Manduca*, both PTTH and insulin stimulate the phosphorylation of Akt in *Bombyx* prothoracic glands, and the activation of PI3K is necessary for ecdysone secretion (Gu et al., 2011a, 2012). Like *Manduca*, MAPkinases are activated by *Bombyx* PTTH, and MEK inhibitors reduce PTTH-stimulated ecdysone secretion (Gu et al., 2010). However, inhibition of PI3K blocks ecdysone secretion even when ERK is activated. Further, insulin can activate the prothoracic glands of *Bombyx* in the absence of ERK phosphorylation (Gu et al., 2009). We have outlined presumptive signaling pathways for PTTH and insulin in lepidopteran prothoracic glands in Figure 9. It appears that in *Bombyx*, unlike *Manduca*, stimulation of PI3K and TOR, through the action of either PTTH or insulin, are requisite steps in ecdysone secretion.

Our results point strongly to an essential role in *Manduca* for the MAPkinase pathway in ecdysone secretion. This is schematized in Figure 9, in which only ERK activation directly stimulates *Manduca* ecdysone secretion, and activation of PI3K and Akt are not required for ecdysone secretion. In *Bombyx*, in which insulin stimulates ecdysone secretion in an apparent absence of ERK activation, it appears that the insulin signaling pathway, including PI3K activation, can stimulate ecdysone secretion without a requisite role for ERK.

In *Manduca*, PTTH enhances the phosphorylation of 4EBP, which is strong evidence for PTTH-stimulated activation of TOR. Previous studies have shown that *Manduca* PTTH-stimulated ecdysone secretion is sensitive to the TOR inhibitor rapamycin and that PTTH enhances the phosphorylation of ribosomal S6 kinase (Song and Gilbert, 1994, 1995). In the present study, phosphorylation of 4EBP was one of the few PI3K-sensitive effects of PTTH (blocked by LY294002). Hence, it appears that PTTH activates TOR through PI3K, yet we did not observe concomitant phosphorylation Akt which would be expected to occur upstream of TOR activation. Also, the phosphorylation of 4EBP could be completely inhibited without affecting ecdysone secretion, indicating that this particular downstream effect of TOR is not essential for acute ecdysone secretion. PTTH enhances the phosphorylation of RSK, and enhanced activation of TOR by RSK has been found in several systems, an effect resulting from its effects on upstream proteins in the TOR pathway (Romeo et al., 2012). However, because 4EBP is phosphorylated in the prothoracic glands even following MEK inhibition, which blocks the phosphorylation of RSK, it is unlikely that RSK serves as the sole TOR activator in *Manduca* prothoracic glands. Hence the mechanism by which *Manduca* PTTH activates TOR remains to be determined.

The reasons for direct insulin stimulation of ecdysone secretion in the prothoracic glands of *Bombyx*, vs. the relative insulin-refractoriness of *Manduca*, are unclear. *Manduca* larvae, like other lepidopteran larvae, are active feeders. Hence there is no a priori reason to expect relative insensitivity of *Manduca* ecdysone secretion to insulin. The difference relative to *Bombyx* may arise in the timing of prothoracic gland growth, which in *Bombyx* occurs relatively late in the last instar (Gu and Chow, 2005). It may be that *Manduca*, in which prothoracic glands double in size daily during the first 4 days of the last instar as larvae feed (Smith, 1995), may dissociate insulin-stimulated glandular growth from secretion to prevent premature metamorphosis. The dissociation of insulin-stimulated glandular growth from acute ecdysone secretion would ensure the proper timing of metamorphosis-inducing ecdysone secretion.

The results of this study are, overall, a reminder that developmental signaling pathways, while fundamentally similar, vary in detail among model insect species. Differences, for example, in the relative impact of PI3K and ERK on ecdysone secretion have presumably evolved to permit different species to optimize the timing of key developmental changes with a conserved toolbox of environmentally and nutritionally sensitive hormonal cues.

## Author contributions

Wendy A. Smith conducted experiments, analyzed data, wrote the manuscript. Anthony Lamattina assisted with experiments, manuscript preparation, and data analysis. McKensie Collins assisted with experiments and data analysis.

### Conflict of interest statement

The authors declare that the research was conducted in the absence of any commercial or financial relationships that could be construed as a potential conflict of interest.

## Abbreviations

4EBP: binding protein for initiation factor 4E
Akt: protein kinase B/Akt
BE: brain extract
cAMP: cyclic adenosine monophosphate
DMSO: dimethyl sulfoxide
ERK: extracellular signal-regulated kinase
GRB2: growth factor receptor-bound protein protein 2
HRP: horseradish peroxidase
IGF: insulin-like growth factor
IR: insulin receptor
LY: LY294002, PI3K inhibitor
MAPK: mitogen-activated protein kinase
MEK: MAP kinase kinase
PAGE: polyacrylamide gel
PI3K: phosphatidylinositol-3-kinase
PLC: phospholipase C
PTEN: phosphatase and tensin homolog, a lipid phosphatase
PTTH: prothoracicotropic hormone
RAF: rapidly activated fibrosarcoma (MAP kinase kinase kinase)
RAS: rat sarcoma protein
RSK: ribosomal S6 kinase
SDS: sodium dodecyl sulfate
SL0101: RSK inhibitor
SOS: son of sevenless protein
StAR: steroidogenic acute regulatory protein
START1: putative cholesterol transporter
TOR: target-of-rapamycin
TRS: Torso receptor substrate
U: U0126, MEK inhibitor.
